# Long-Term Assessment of the Properties of Load Sensors Applied in Weigh-in-Motion Systems

**DOI:** 10.3390/s25082421

**Published:** 2025-04-11

**Authors:** Janusz Gajda, Ryszard Sroka, Piotr Burnos, Mateusz Daniol

**Affiliations:** Department of Measurement and Electronics, AGH University of Krakow, Al. A. Mickiewicza 30, 30-059 Kraków, Poland; jgajda@agh.edu.pl (J.G.); burnos@agh.edu.pl (P.B.); daniol@agh.edu.pl (M.D.)

**Keywords:** dynamic weighing of road vehicles, weigh-in-motion (WIM) systems, direct mass enforcement, stationarity of measurement systems, long-term assessment of accuracy

## Abstract

The noticeable growth of road transport means that the protection of road infrastructure is becoming a critical issue. The main factor leading to the excessive degradation of roads are overloaded vehicles. The effective elimination of such vehicles from road traffic is possible through widespread usage of Weigh-In-Motion (WIM) systems for direct mass enforcement, thus eliminating the need for “manual” vehicle checks which are currently carried out by the appropriate services. WIM mass enforcement systems require strict metrological control, meaning that an initial verification, conducted at the moment when the system is installed, and subsequent periodic verifications are required. These operations aim to ensure that vehicle weighing error is consistently maintained within a permissible range of values. Fulfilment of this condition allows for the minimisation of the probability that a vehicle loaded within normative limits will be classified as overloaded. The long-term study of two WIM systems located on provincial road 975 in Wielka Wies, in southern Poland, equipped with load sensors made using different technologies (strain gauge sensors and quartz sensors) and in different weather conditions, has allowed us to formulate recommendations regarding the frequency with which subsequent verifications should be performed in order to ensure the reliability of the weighing results. This paper presents the results of these studies and conclusions formulated based on them; in this case, they showed a verification of the system can be performed every 8 months. The conclusions and recommendations that we have presented concern primarily those WIM stations which were the object of our study and caution should be exercised when generalising these to other cases. Its novelty results from several premises. For the first time, long-term studies of two WIM systems equipped with load sensors made with different technologies were carried out. Both systems were installed on the same surface, in the immediate vicinity of each other. They were installed on a standard road and were subjected to the constant impact of road traffic with identical parameters. Tests of both WIM systems were performed periodically, using the pre-weighed vehicles method, in different seasons, for a period of 15 months. During the tests, the same test vehicles drove through both WIM systems at the same speed. All of this resulted in the obtainment of a unique set of measurement data, the analysis of which allowed for the assessment and comparison of the proprieties of the load sensors made with both technologies.

## 1. Introduction

Efforts to provide effective protection for road infrastructure have resulted in an intensive study of the structure and properties of Weigh-In-Motion (WIM) systems for direct mass enforcement. The negative impact of overloaded vehicles has been addressed in an opinion formulated by the American Association of State Highway and Transportation Officials (AASHTOs), stating that the passage of a single heavy vehicle with a mass of 20 tons causes damage to road infrastructure comparable to the passage of 20,000 passenger cars [1,2].

The basic purpose of using WIM systems is to eliminate overloaded vehicles from traffic. WIM systems provide rich measurement information on road traffic in a specific location. For this reason, they are also used for other purposes, such as road infrastructure management, repair planning, the design of infrastructure elements, and fatigue analyses of elements such as motorways or bridges. The measurement results of parameters such as traffic intensity, vehicle position, speed, distance between vehicles, total weight, and axle load are the basis on which models describing the traffic on the analysed infrastructure object are built. These models allow for predicting the fatigue life of this infrastructure element [3,4]. In some cases, the measurement information provided by WIM systems is insufficient. Then, the solution to the problem is sought by building adequate traffic models, which, among other things, use data from the WIM system [5].

**Figure 1:**
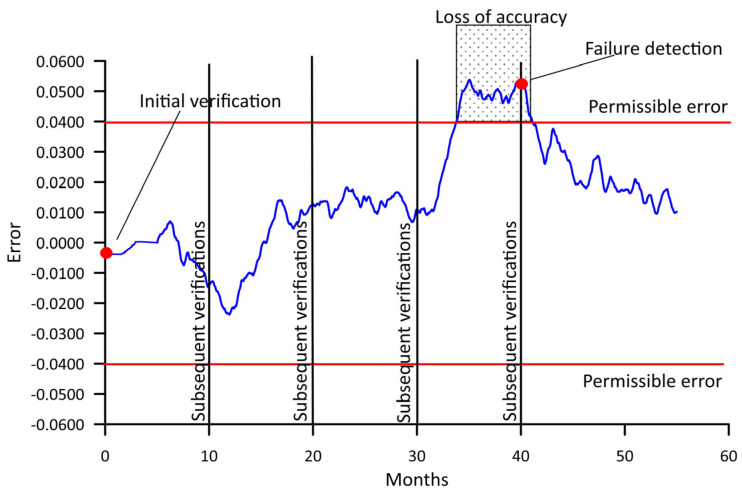
Illustration of the variability of accuracy of vehicle weighing in WIM systems, developed on the basis of [8].

Currently, documentation is being developed defining procedures for the metrological legalisation (verification) of such systems [6,7]. In neither of the cited documents, however, can we find any recommendations on the frequency of repetition of the subsequent verifications. This problem is illustrated in Figure 1.

In paper [8], the authors draw attention to the problem of lack of information concerning the accuracy of WIM systems in the periods between subsequent verifications. They propose that a subsequent verification be conducted one year after the installation of the WIM system and after the initial verification. They also propose the implementation of a combination of three measures as a method for ensuring the reliability of the weighing results in the period between verifications: continuous checks, maintenance, and calibration. In document [9], it is stated that In-Service Verification should be performed at least once a year. However, the authors stress that this recommendation refers to the statistical application of WIM systems. The document does not refer to this issue in the context of WIM systems for direct mass enforcement.

The initial and subsequent verifications of WIM systems aim to ensure that the vehicle weighing error is consistently maintained within a permissible range of values. Thus, they should ensure that a normative vehicle is not classified as overloaded. The metrological verification of WIM systems is conducted after the installation of the system, constituting the initial verification or an acceptance test. Next, this verification is repeated with a set frequency, known as the subsequent verifications. The documents cited above either fail to indicate the time interval after which a subsequent verification should be conducted completely or suggest verification during a single year. Due to the time-consuming nature and costliness of the verification of WIM systems, there is a natural tendency to try to prolong the periods after which a subsequent verification is conducted. Nevertheless, a number of diverse factors impact the accuracy of WIM systems: the mechanical parameters of the road and its degradation over time, the technical parameters of the weighed vehicles (tire type, tire pressure, suspension type, etc.), and among these, the most significant is environmental factors (temperature, precipitation, wind, etc.) [10,11,12,13]. The impact of these factors is both short-term (the changes seen over the course of a day) and long-term (observed in successive seasons of the year). Furthermore, over longer periods of time, changes in weighing accuracy may occur as a result of the degradation of the pavement in which the sensors are installed. The process of the “ageing” of the load sensors and the resulting loss of sensitivity can also be seen, as well as changes in the parameters of the electronic measurement systems working together with the sensors visible during a change in amplification (the slope of the static characteristic) or zero drift (offset).

In the available literature, there is no information on how fast these processes occur, yet such knowledge is necessary to establish the required frequency of repetition of subsequent verifications. The present paper is an attempt to answer this question. The basis for the formulation of the answer are the results of a 15-month experimental study of two WIM stations, working in continuous operation mode in standard road traffic conditions. These systems were equipped with load sensors made using different technologies. The analysis of the collected measurement results allowed for the determination and justification of a required frequency of repetition of subsequent verifications in WIM systems for direct mass enforcement.

The metrological verification of WIM systems is generally conducted using one of two well-known and currently implemented methods. Both methods involve a comparison of the weighing results of selected test vehicles obtained from the WIM system, with the weighing results of these same vehicles on static reference scales and/or on low-speed (LS-WIM) scales. The reference scales must have valid metrological certificates determining their required accuracy class.

The first method is the pre-weighed vehicle method [14]. The pre-weighing of selected test vehicles on reference scales allows for the determination of their gross vehicle weight (GVW) and the static load of each individual axle. Next, the test vehicles make multiple runs through the WIM station. The weighing error is determined as the difference between the result from the WIM station and the values measured on the reference scales. The error of GVW weighing is also determined, as is the error of the axle load. The number and parameters of the test vehicles and the metrological parameters of the reference scales are set out in the relevant documents [15].

The second method involves the weighing of vehicles drawn from the flow of traffic at the WIM station. After completing a run over the WIM station, these vehicles are then directed to reference scales which should be located nearby [16]. The required number of results to be collected depends on the accuracy of the calibrated WIM station [17]. Due to the uncertainty of the estimation of the coefficients of the static characteristic of the WIM station and to the desire to minimise this uncertainty, in the cited paper, it was established that to ensure the accuracy of the calibration at the level of 1%, it was necessary to perform 400 test vehicle runs. This is an indication of how time-consuming and costly this calibration process can be for a WIM system of the highest accuracy class. Both methods are described in detail in ASTM Standard E1318-02 [18,19].

As can be seen from the presented description of the calibration verification methods used for WIM systems, they are quite complex in logistical terms, time-consuming, and costly. The logistical complexity results from the need to have access to test vehicles of the required axle configurations and reference scales located in the near vicinity of the WIM system being calibrated.

The time required for these methods results from the fact that the entire procedure is performed on a typical road in which the WIM system is installed, in normal road traffic conditions. The test vehicles must be able to turn around and loop back to their starting point. Finding an appropriate and safe place for this manoeuvre in many cases means driving several dozen kilometres or more. Additionally, the drivers of the test vehicles are limited to an 8-hour work day with a break in the middle. Thus, the calibration of a single WIM system can occupy an entire day.

The problem of the lack of stationarity in WIM systems could be lessened by the implementation of autocalibration algorithms in the system. This solution is known and has been described in the literature [20,21,22,23,24]. The idea of this method is relatively simple and is based on the observation that the load of a selected axle of a heavy, multi-axle vehicle is relatively poorly correlated with GVW. Vehicles like these are called characteristic vehicles. This means that the load of this selected axle can be treated as a reference value with known static parameters. Simultaneously, the detection of a characteristic vehicle at a WIM station is simple and involves the specific and known configuration of the vehicle’s axles and their number. Each run of the characteristic vehicle through the WIM station can be used to correct the parameters of the static characteristic of the weighing system. The way in which the weighing results of characteristic vehicles is used depends on the autocalibration algorithm implemented.

It should be stressed that autocalibration is not a substitute for the verification of a WIM system for mass enforcement, but it can extend the period over which such verification must be repeated. This topic has not yet been the subject of research as of today.

If the logistical and time needs described above are multiplied by a dozen or many dozens of WIM systems requiring periodic calibration, then there is good reason to attempt to reduce the frequency with which subsequent verifications must be performed. The question of to what extent this frequency can be reduced without causing an excessive increase in weighing error thus arises. The minimum value of this frequency is dependent on the degree of the non-stationarity of a WIM system. Of course, the determination of this minimum frequency does not eliminate the problem of critical events for the operation of the WIM system (e.g., damage occurring to some of its elements), which must be followed by a subsequent verification regardless of the timetable.

The aim of the studies conducted for this paper was to search for answers to the questions outlined above regarding the minimum permissible frequency for repetition of the subsequent verifications of WIM systems for mass enforcement.

The paper is organised as follows: in Section 2, we present the research methodology. This section includes descriptions of the two WIM stations at which the experiments were conducted. It also includes a discussion of the parameters of the road and pavement in which the load sensors were installed. Additionally, we propose a reliability characteristic of WIM systems and the error δ_0.95_ as a measure of the accuracy of the studied WIM systems. Section 3 includes a description of the experiments, in particular, the number, class, and parameters of the test vehicles used and their number of runs through the WIM stations; the types and parameters of the reference scales used; the periods during which the experiments were conducted; and the prevailing temperature conditions. Section 4 includes the measurement results, and the parameters and metrological characteristics for both WIM stations determined on their basis. Section 5 presents conclusions resulting from this study, with particular attention paid to the scope of the weighing error occurring in the periods between individual experiments, with differentiation into random errors and systematic errors. In the summary, we formulate a recommendation on the permissible length of the period between subsequent verifications of these systems.

## 2. Methodology

This chapter presents the research stand where the experiments described in the paper were carried out. The studies were conducted at two WIM stations which were each equipped with four lines of load sensors, with two sensors in each line [25].

### 2.1. Sensor Arrangement

**Figure 2:**
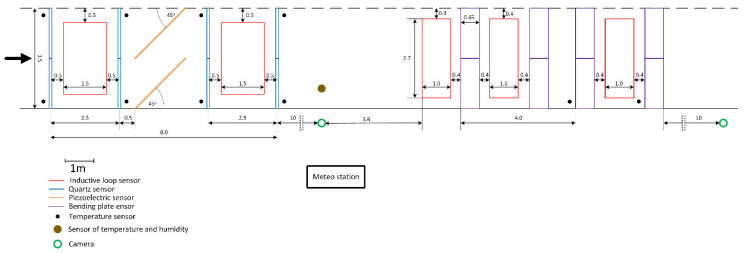
Diagram of two WIM stations equipped with four lines of quartz load sensors and four lines of bending plate load sensors, respectively.

**Figure 3:**
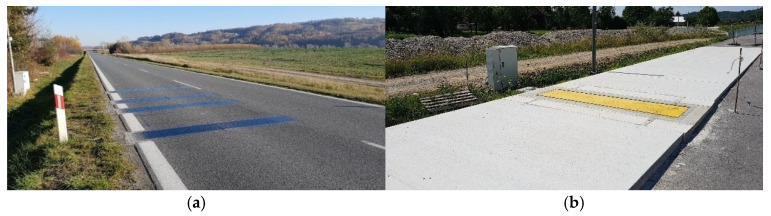
WIM station equipped with four lines of bending plate sensors (**a**) and a low-speed LS-WIM station (**b**), TENZOVAHY, Olomouc, Czech Republic.

The first station was equipped with quartz load sensors (Kistler GmbH, Wien, Austria), and the second with bending plate load sensors, Saskatoon, SK, Canada (Figure 2). Such a configuration of load sensors facilitates the independent, fourfold measurement of the load of the left and right wheel of each axle. By summing up the measurement results of the load of each wheel, the result of the axle load measurement can be determined separately for each line of sensors. It is also possible to calculate the arithmetic mean for two, three, or four lines of sensors. Between the load sensors, induction loops and diagonal piezoelectric sensors were installed, which made it possible to monitor the correct placement of the weighed vehicle. Figure 3a shows a fragment of the weighing station equipped with bending plate sensors.

Both WIM stations were additionally equipped with a set of sensors allowing for the measurement of environmental factors. This made it possible to study the effect of these factors on the results of vehicle weighing. It was possible to measure the force and direction of the wind, to detect precipitation, to measure moisture levels on the road surface or flooding of the pavement, as well as salt levels on the road surface. A set of temperature sensors measured the temperature in the substructure of the road to a depth of 30 cm with a spatial resolution of 5 cm, the temperature of the pavement at a depth of 0 cm and −5 cm at the ends of the load sensors, as well as the air temperature. Four installed cameras made it possible to observe road traffic at the WIM station and to recognise and record the registration numbers of the weighed vehicles.

### 2.2. Pavement Quality Parameters

The parameters characterising the pavement quality at the site where the WIM stations were installed include curvature of the road, lateral and longitudinal inclination, unevenness of the road surface in the scope of high and low values of spatial frequency, as well as road deflection in static and dynamic conditions. Land surveys were conducted to determine the lateral and longitudinal inclination. The difference in levels over a distance of roughly 150 m was less than 100 cm, meaning that the longitudinal inclination is at a level below 0.7%. Lateral inclination was 2% in the direction of the road shoulder. Measurements of the unevenness of the pavement were performed with a laser profilometer [26,27]. The roughness of the pavement was assessed used the IRI index [27], whose mean value for a Class A road should not exceed 1.3 mm/m, with a maximum value of 2.4 mm/m. In the place where the sensors were installed at the WIM stations, these values were 1.1 mm/m and 2.0 mm/m, respectively. Measurements of the deflection of the pavement were performed using the FWD method [14]. At none of the measurement points did the deflection exceed the value of 223 µm. Deflection measurement results in the range of 150–250 µm will classify this road surface as “good” [28].

### 2.3. Experimental Program

In the immediate vicinity of both WIM stations, LS-WIM scales were constructed (Figure 3). These scales possess metrological verification and serve as reference scales for the verification of the correctness of weighing results at the WIM stations.

Tests of both WIM stations were conducted using the pre-weighed vehicles method [18]. This method involves multiple runs through the WIM station by test vehicles which were previously weighed in static or quasi-static conditions on a platform scale and a low-speed WIM scale or on portable scales. All scales should be characterised by a known accuracy, confirmed by valid metrological verification. Measurement on the platform scale allows a reference value of the gross vehicle weight (GVW) to be determined for each test vehicle. The LS-WIM scale, over which the weighed vehicle passes with the speed restricted to roughly 5 km/h, allows reference values for the load of each axle to be determined. An alternative method is to weigh the test vehicles on portable scales placed simultaneously under all of the vehicle’s wheels on a level site. Such a measurement allows both for the determination of a reference GVW value for the test vehicles and also for reference values of static load of all the wheels/axles of these vehicles.

The aim of the test performed was to collect measurement data allowing for an assessment of the stationarity of both WIM stations. By this, we mean the change in the accuracy of the weighing results observed as a function of time. However, the observed change in accuracy may also be caused by other factors, e.g., temperature, also changing as a function of time. Taking this aim into consideration, this test was performed five times over the course of 15 months. In detail, it was performed in April 2023, November 2023, December 2023, April 2024, and July 2024. On the basis of the collected data, weighing errors for the tested vehicles occurring at both WIM stations were determined for the periods listed.

### 2.4. Data Analysis

Vehicle weighing errors at the WIM stations included both a systematic component and a random component. The causes of these two types of error are various. A systematic error is caused primarily by changes in the coefficients of the static characteristic of a WIM station (slope and offset). This error can be alleviated through the calibration of the WIM station and the adjustment of the coefficients of its characteristic. However, as these coefficients change over time (non-stationarity), an effective reduction of the systematic weighing error component may require repetition of the calibration procedure. Even with this, in the periods between successive calibrations, the occurrence of a systematic error must by accounted for.

Random errors are caused primarily by vertical oscillations of the weighed vehicle during its run through a WIM station. In reality, it is the dynamic load of the individual wheels that is measured directly, and, on this basis, the static load is estimated. The intensity of the dynamic load component depends on the state of the pavement, the speed of the weighed vehicle, its construction, and the type and parameters of its suspension. A second cause of random error is the heterogeneity of load sensors indicated by changes in their sensitivity as a function of length, in combination with the random choice of trajectory by a given vehicle. Random events such as, for example, the speed of the vehicle or random gusts of wind must also be taken into account. Additionally, after the elimination of the systematic error, there remains a component caused by the uncertainty of the calibration process and the adjustment of the coefficients of the static characteristic.

If a WIM station has been properly calibrated, it can be assumed that the systematic errors have been minimised and that the weighing errors will be dominated by random errors. Assuming this, statistical measures are applied for the assessment of the accuracy of the WIM station, e.g., the error δ_0.95_. This is the value of the relative error δ which fulfils the following condition: *Probability*(δ ≤ δ_0.95_) = 0.95, meaning that 95% of the weighing results are determined with an error no greater than δ_0.95_. The statistical interpretation of the error δ_0.95_ can be easily illustrated by using the reliability characteristic determined for the studied WIM station.

The idea of using reliability characteristics for the assessment of the accuracy of WIM stations was proposed by the authors of this paper in [29], and subsequently developed in [30,31].

The reliability characteristic is described in the dependence (1). The basis for determining the reliability characteristic is the statistical analysis of the experimental results obtained using the pre-weighed vehicles method. The reliability characteristic determines the probability (*Pr*) of the occurrence of a weighing error with a value greater than the value of the argument of the characteristic |*x*|. The error δ_0.95_ is thus a value of the argument |*x*| for which the reliability characteristic attains a value of 0.05. An additional benefit of using the reliability characteristic is that it allows for an easy visual comparison of the two stations in terms of their accuracy. The station whose reliability characteristic decreases faster can be understood to be more accurate, as this means that errors of greater values are less probable.

*Pr*(|*x*|) = 1 − *Φ*(|*x*|) (1)

where |*x*| is the absolute value of the measurement error, and *Φ*(·) is the estimate of the cumulative distribution function of the variable |*x*|.

## 3. Description of Experiments

The experiments were conducted at a field measurement site located on provincial road No. 975 in Wielka Wies, southern Poland. The exact coordinates of the site are as follows: 49° 55′ 35″ N, 20° 50′ 09″ E.

During the experiments conducted, a set of vehicles was used on each occasion including the following: a two-axle rigid vehicle, a three-axle vehicle, and a five-axle vehicle comprising a two-axle tractor with a three-axle semitrailer (2T3S). The parameters of the test vehicles used in the successive testing periods are presented in Table 1.

Over the course of the experiments, a total of 377 test runs were performed with all of the test vehicles run through the studied WIM stations. A detailed breakdown of the number of runs for individual test vehicles is presented in Table 2. The test vehicles passed through the studied stations at a steady speed between 50 km/h and 80 km/h. The drivers were instructed to maintain a steady speed and a straight trajectory throughout the run through of the WIM station.

**Figure 4:**
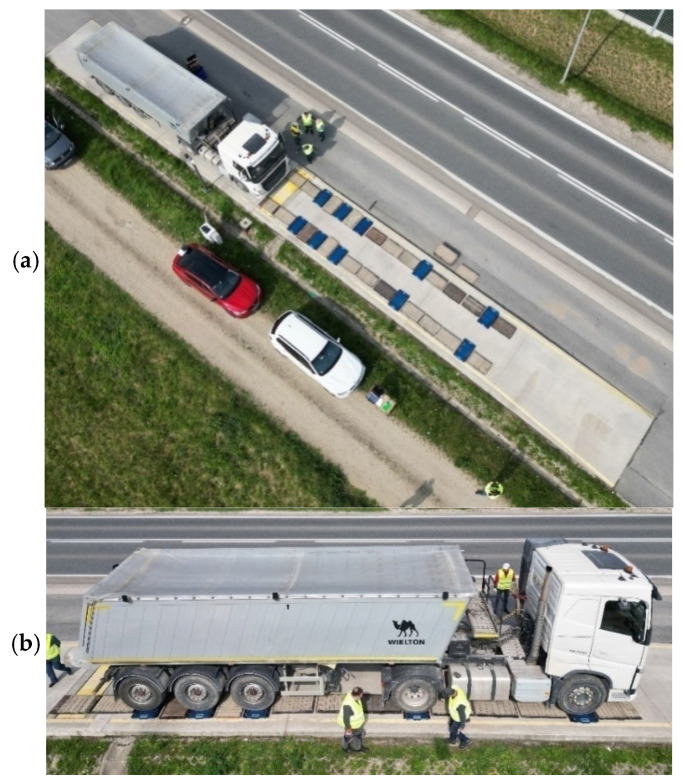
Weighing a 5-axle vehicle on portable scales, (**a**) arrangement of scales, and (**b**) weighing in progress.

The test vehicles were pre-weighed on a platform scale and on a low-speed scale or portable scale, depending on availability. The arrangement of the five-axle vehicle weighing station on portable scales is shown in Figure 4.

The metrological parameters of the vehicle scales used are shown in Table 3. The errors of the studied WIM stations were compared on each occasion to the GVW reference values and to the axle load determined using a low-speed (LS-WIM) scale.

The weighing of test vehicles was performed at various times of the year, and thus in a variety of weather conditions. The maximum air temperature values were recorded on the days on which the experiments were conducted and are presented in Table 4.

## 4. Measurement Results

**Figure 5:**
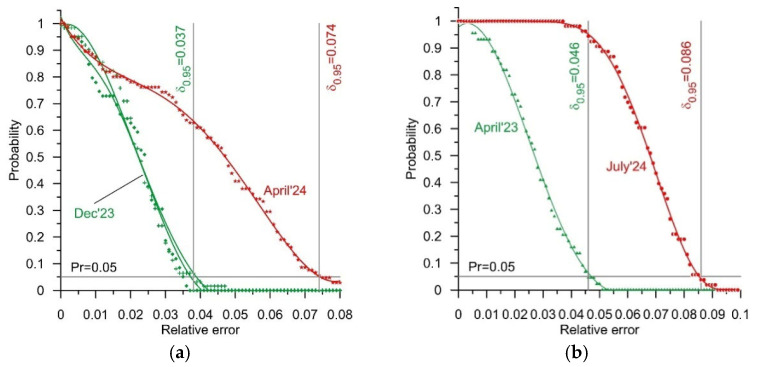
Reliability characteristics for extreme values of the error δ_0.95_, for GVW measurements at the WIM station equipped with four lines of bending plate sensors (**a**) and the WIM station with four lines of quartz sensors (**b**).

**Figure 6:**
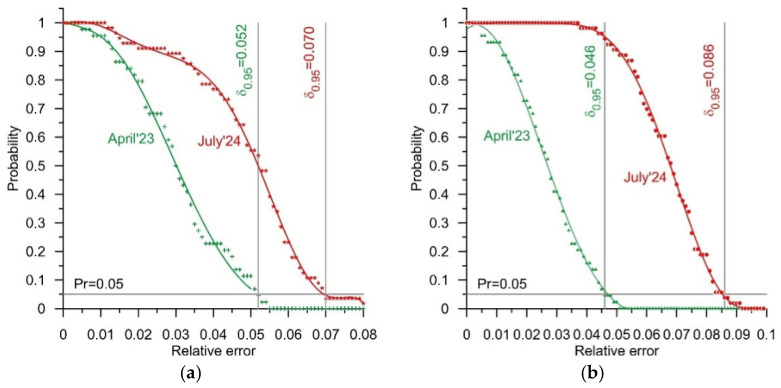
Reliability characteristics for extreme dates of the conduct of the experiments, for GVW measurements at the WIM station equipped with four lines of bending plate sensors (**a**) and the WIM station with four lines of quartz sensors (**b**).

The measurement results for the test vehicles, obtained in April, November, and December of 2023, and April and July of 2024, were the basis for the determination of reliability characteristics (1). We determined such characteristics for both of the WIM stations, i.e., for the station equipped with bending plate load sensors and for the station equipped with quartz sensors. These characteristics illustrate the probability of an occurrence of a determined value of relative error in a GVW measurement as a function of the value of this error. The GVW measurement result was set as the arithmetic mean of the weighing results obtained at each of the four lines of load sensors comprising the studied WIM station. The selected reliability characteristics are presented in Figure 5. These were chosen so as to reflect the extreme values of the error δ_0.95_ observed during the successive experiments. Furthermore, in Table 5, the values of the error δ_0.95_ determined on the basis of the weighings conducted during all of the experiments are collated. In Figure 6, in turn, the reliability characteristics of both systems are presented, reflecting the extreme dates at which the experiments were conducted, that is April 2023 and July 2024. In the case of the WIM system equipped with quartz load sensors, these are the same characteristics which were presented in Figure 5b. In this case, the extreme values of error occur at the extreme dates at which the experiments were conducted.

The characteristics presented in Figure 5 and Figure 6 and the results presented in Table 5 lead to a very positive conclusion. Both WIM systems were studied over a period of 15 months. During this time, they operated in normal road traffic conditions and were not subject to any technical interventions. Despite this, in the case of both WIM stations, the changes in the value of the error δ_0.95_ observed over the period of April 2023–July 2024 were confined within a range of 0.04 (4%). The distribution of the errors also changed. This is especially visible in the case of the system equipped with quartz sensors. During the experiments conducted in July 2024, an occurrence of errors of less than 0.04 (4%) was not observed. The reliability characteristic (Figure 5b) was flat in the range of error values from 0 to 0.04. The maximum weighing error increased to 0.086 (8.6%); yet, the width of the range of variability of this error was still roughly 0.04 (4%). This means that after an adjustment of the static characteristic of the WIM system (system calibration), the GVW measurement error remained within a range of +/−0.02 (+/−2%). This is a very good result.

**Figure 7:**
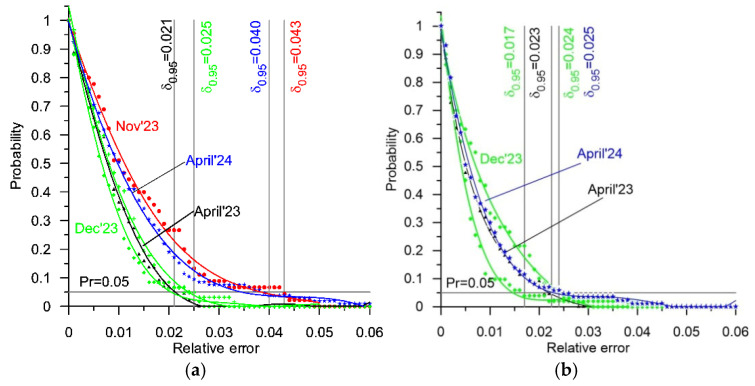
Reliability characteristics after adjustment of static characteristic for GVW measurements at the WIM station equipped with four lines of bending plate sensors (**a**) and the WIM station with four lines of quartz sensors (**b**).

The causes of weighing errors are highly diverse. They include first and foremost the vertical oscillations of the weighed vehicle during its run through a WIM station. The errors caused by this phenomenon are of a random nature. A second cause of errors is the impact of atmospheric conditions, including primarily the impact of temperature. Considering that each of the experiments was conducted over the course of a single day (roughly 8 hours), it can be assumed that the errors caused by these phenomena are of a systematic nature. A third cause of errors are changes as a function of time in the metrological properties both of the sensors and of the electronic measuring systems which work together with the sensors. These involve changes in sensitivity, changes in amplification, and shifts in the zeroes of the static characteristics. Limiting the impact of this phenomenon on the accuracy of a WIM system requires periodic calibration and an adjustment of the parameters of its static characteristic. This hypothesis is confirmed by the characteristics presented in Figure 7. These are example reliability characteristics of both the studied WIM systems, determined after an adjustment of the parameters of their static characteristics.

Table 6 presents collated values of the error δ_0.95_ of the GVW measurements after an adjustment of the static characteristics of both WIM systems.

In the case of the WIM station equipped with bending plate sensors, the maximum difference between the values of the error δ_0.95_ after the adjustment of the parameters of the static characteristic was roughly 0.02 (2%). The collected measurement data do not allow for an authoritative explanation of the causes responsible for the occurrence of this difference at the present stage of research. This is especially so as it does not change in a monotonic manner as a function of time.

Such changes did not occur at the station equipped with quartz sensors. The greatest difference between the values of the error δ_0.95_ after an adjustment of the parameters of the static characteristic was 0.008 (0.8%). Considering that this concerns the errors of vehicle weighing performed in standard road traffic conditions and at normal road traffic speeds, the statement that the characteristics determined during various periods were nearly identical is justified.

The correct conduction of the experiments and analysis of the data are confirmed by the characteristics determined in December 2023. The experiment conducted at that time lasted two days, using the same test vehicles. The reliability characteristics determined based on the measurement data obtained separately on each day were nearly identical.

The characteristics presented in Figure 7 also allow for the formulation of conclusions concerning weighing accuracy. The maximum value of error δ_0.95_ after an adjustment of the static characteristic was 0.043 (4.3%) for the station equipped with four lines of bending plate sensors and roughly 0.025 (2.5%) for the station equipped with quartz sensors. These are the maximum values for the errors δ_0.95_ occurring at these stations during the conduction of the tests. To a large extent, they are of a random nature (systematic errors were eliminated as a result of the adjustment performed). The causes of random errors are various. First and foremost, they are caused by vertical oscillations of the weighed vehicle during its run through a WIM station. The amplitude of these oscillations is dependent on the state of the pavement and the construction parameters of the vehicle’s suspension as well as its speed. The remaining causes of random errors are the heterogeneity of the load sensors indicated by changes in their sensitivity as a function of length and the uncertainty of the adjustment of the parameters of the static characteristic, as well as the manner in which the vehicle crosses the measurement station.

The conclusions formulated on the basis of the reliability characteristics presented above (Figure 5, Figure 6, and Figure 7) confirm the observed changes in the coefficients of the static characteristics of both WIM stations. The values of these coefficients determined for each testing date are presented in Table 7.

In the case of both WIM stations, both changes in the slope of the static characteristic and changes in the offset were observed. The scopes of these changes are comparable in the cases of both of the stations; however, they occurred at different times.

**Figure 8:**
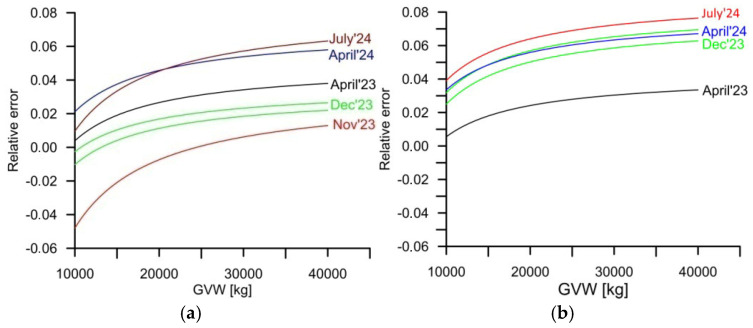
Dependence of relative error of the static characteristic of WIM with reference to the ideal characteristic, depending on the measured GVW value: (**a**) WIM station equipped with bending plate sensors, and (**b**) WIM station equipped with quartz sensors.

The share of weighing errors caused by changes in the parameters of the static characteristic in the total uncertainty budget of GVW weighing errors at the WIM station equipped with four lines of load sensors is illustrated by the characteristics presented in Figure 8a. These were calculated as the relative difference between the actual static characteristic and the ideal characteristic, for which the *slope* = 1.0 and *offset* = 0.0. This difference was calculated in a GVW range of 10 t to 40 t and applied to the actual measured value.

The weighing error described by the characteristics presented in Figure 8 is of a systematic nature. For the station equipped with bending plate sensors, this ranged from −5% to +5%. In the case of the station equipped with quartz sensors, this range was from −0.5% to 7%. This error can be eliminated with a sufficiently frequent adjustment of the coefficients of the static characteristic. Assuming that such an adjustment is performed no more frequently than every 15 months, and the total (maximum random + systematic) error of the GVW measurement of five-axle vehicles at a system equipped with four lines of load sensors should not exceed 0.11 (11%), comprising 0.065 (6.5%) systematic error and 0.043 (4.3%) random error for bending plate sensors. In the case of the WIM system equipped with quartz load sensors, the maximum GVW measurement error should not exceed 0.095 (9.5%), comprising 0.07 (7%) systematic error and 0.025 (2.5%) random error.

**Figure 9:**
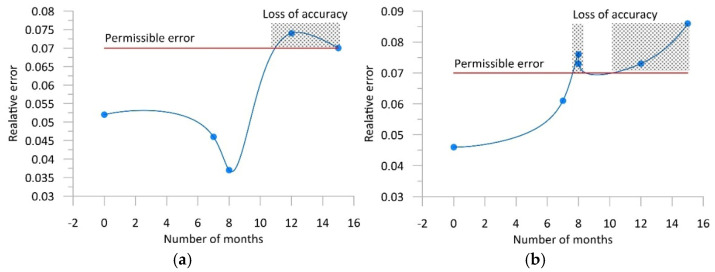
Variability of the error δ_0.95_ of the GVW measurements as a function of time. (**a**) WIM system equipped with bending plate sensors, and (**b**) WIM system equipped with quartz sensors. The solid line is merely the result of an approximation of the measurement points.

Figure 9, referring back to Figure 1, presents the variability of the error δ_0.95_ as a function of time for both WIM stations. Assuming that the maximum permissible value of GVW measurement error is 0.07 (7%), reflecting an accuracy class of B+(7), it can be determined when this error exceeds the assumed limit, and it is necessary to perform a calibration of the system and an adjustment of its static characteristic.

It is possible to achieve an increase in this accuracy by performing more frequent adjustments of the static characteristic of a WIM station or by implementing an autocalibration algorithm [21,22,23]. The minimum weighing error values achievable for both WIM stations result from the characteristics presented in Figure 7, amounting to 4.3% for the station equipped with bending plate sensors and 2.5% for the station equipped with quartz sensors.

## 5. Conclusions

In total, 377 runs of test vehicles belonging to different classes were performed. These runs were performed at various speeds within the range of 50 km/h–80 km/h. They were performed at various times of the year, and thus in various weather conditions. The experimental results described above allow for the formulation of the following conclusions:

- After nearly three years in service (their calibration was conducted during the initial setup), both systems exhibited a change in their static characteristic slopes and also a shift in zeroes:
- The change in the static characteristic slope of the system equipped with bending plate load sensors was 4.8%, while for the system equipped with quartz load sensors the change was 4.6%;
- The error δ_0.95_ of the GVW measurement in the bending plate system was 0.052 (5.2%) in April 2023, 0.046 (4.6%) in November 2023, 0.037 (3.7%) in December 2023, 0.074 (7.4%) in April 2024, and 0.07 (7%) in July 2024;
- The error δ_0.95_ of the GVW measurement in the quartz sensor system was 0.046 (4.6%) in April 2023, 0.073–0.076 (7.3–7.6%) in December 2023, 0.073 (7.3%) in April 2024, and 0.086 (8.6%) in July 2024;

When analysing the results obtained, presented in the form of reliability characteristics, attention should also be paid to those characteristics determined after a correction of the weighing results. This correction was performed virtually on each occasion, based on the current static characteristic of each WIM system (Figure 7). These characteristics indicate the fluctuation of random errors which cannot be limited through a correction of the static characteristic. 

## 6. Summary

The experimental results collected from two WIM stations over the course of 15 months and the results of the analysis lead to three key conclusions:

1. The efforts to maintain the systematic component of the weighing error of road vehicles at a level of below 5% require a calibration of the WIM system at least every 8 months.
2. One alternative to the cyclical repetition of the calibration of WIM stations using the pre-weighed vehicles method is the implementation of an autocalibration algorithm.
3. The maximum values of the random error component δ_0.95_ for the tested stations are 0.043 (4.3%) for the station equipped with bending plate load sensors and 0.025 (2.5%) for the station equipped with quartz load sensors. The values cited in this paper represent the lower boundaries of uncertainty for the vehicle weighing results at the studied WIM stations. Obviously, we realise that the conclusions and recommendations that we have presented above concern primarily those WIM stations which were the object of our study. Caution should be exercised when generalising these to other cases.

## Figures and Tables

**Table 1:** Results of the reference weighing of test vehicles on a low-speed scale.

Vehicle Class	GVW [kg]	Ref. Value of Axle Load I [kg]	Ref. Value of Axle Load II [kg]	Ref. Value of Axle Load III [kg]	Ref. Value of Axle Load IV [kg]	Ref. Value of Axle Load V [kg]	Ref. Value of Axle Group [kg]
April 2023							
2-axle	16,240	6298	9942	x	x	x	x
3-axle	25,380	7268	8916	9196	x	x	18,112
5-axle	39,861	7184	11,346	7216	7152	6963	21,331
November 2023							
2-axle	15,916	6472	9444	x	x	x	
3-axle	23,324	7024	8088	8212	x	x	16,300
5-axle	37,364	7076	10,872	6504	6656	6256	19,416
13 December 2023							
2-axle	7984	4644	3340	x	x	x	x
3-axle	22,043	6493	7723	7827	x	x	15,550
5-axle “A”	39,744	7008	11,540	7176	7088	6932	21,196
5-axle “B”	30,220	6540	7600	5388	5408	5284	16,080
14 December 2023							
2-axle	7996	4656	3340	x	x	x	x
3-axle	21,996	6428	7724	7844	x	x	15,568
5-axle “A”	39,692	6956	11,540	7164	7092	6940	21,196
5-axle “B”	30,072	6460	7532	5372	5404	5304	16,080
April 2024							
2-axle	8113	4778	3335	x	x	x	x
3-axle	22,188	7052	7555	7581	x	x	15,136
5-axle	39,794	7105	12,133	6956	6909	6691	20,556
July 2024							
2-axle	167,96	7096	9700	x	x	x	
3-axle	24,060	7544	9864	6652	x	x	16,516
5-axle	38,504	6908	8204	7808	7784	7800	23,392

**Table 2:** Number of runs of test vehicles through the studied WIM station.

	Vehicle Class		
Date of Experiment	2-Axle Vehicle	3-Axle Vehicle	5-Axle Vehicle
April 2023	15	14	15
Nov 2023	15	15	15
13 Dec 2023	15	15	29
14 Dec 2023	15	16	31
April 2024	36	33	36
July 2024	21	21	20
TOTAL 377	117	114	146

**Table 3:** Parameters of vehicle scales used in the conducted experiments.

Item	Type of Scale	Manufacturer	Accuracy Class	Measurement Range [kg]	Scale Interval [kg]	Vehicle Speed [km/h]
1.	DFW portable scale	Dini Argeo	III ^1^	10,000	5 (ext. 0.5) ^2^	N/A
2.	Platform scale	WITWAG	III ^1^	400–60,000	20	N/A
3.	Low-speed LS-WIM scale, type VM 1.2	TENZOVAHY—The Czech Rep.	D2 ^3^	400–20,000	20	1–6

^1^ This class concerns the so-called non-automatic scales (usually industrial scales), their accuracy is described by the value of the verification scale interval—in this case, ±20 kg (this value depends on the range of the scale and maximal number of intervals). ^2^ The abbreviation *ext.* means that the scale is an extended displaying device. Such a device temporarily changes the actual scale interval to a value less than the verification scale interval following a manual command. ^3^ Accuracy class D2 means that the gross weight of the vehicle is indicated with an error no greater than 2%, and that the static individual axle load is indicated with an error no greater than 4% [15].

**Table 4:** Maximum air temperature values for the dates when the experiments were conducted.

	Maximum Air Temperature [°C]	
Date of Experiment	Day	Night
April 2023	17	5
Nov 2023	3	−9
13 Dec 2023	5	1
14 Dec 2023	3	2
April 2024	17	7
July 2024	29	20

**Table 5:** Error values δ_0.95_ determined based on the results of the weighing of road vehicles obtained during successive experiments.

Date of Experiment	Error δ_0.95_	
	Bending Plate Sensors	Quartz Sensors
April 2023	0.052	0.046
Nov 2023	0.046	no data
13 Dec 2023	0.037	0.076
14 Dec 2023	0.037	0.073
April 2024	0.074	0.073
July 2024	0.070	0.086

**Table 6:** Error values δ_0.95_ determined based on vehicle weighing results obtained during successive experiments after adjustment of the static characteristics of both WIM systems.

Date of Experiment	Error δ_0.95_	
	Bending Plate Sensors	Quartz Sensors
April 2023	0.021	0.023
Nov 2023	0.043	no data
13 Dec 2023	0.025	0.017
14 Dec 2023	0.025	0.024
April 2024	0.040	0.025
July 2024	0.023	0.018

**Table 7:** Coefficients of the static characteristics of the studied WIM stations at subsequent dates of measurements, with reference to the results of weighing on a low-speed WIM scale.

	Bending Plate Sensors		Quartz Sensors	
	Characteristic Slope	Characteristic Offset [kg]	Characteristic Slope	Characteristic Offset [kg]
April 2023	1.0494	−454.6	1.0429	−373.1
Nov 2023	1.0332	−812.2	no data	no data
Dec 2023	1.0362	−387.7	1.0820	−501.5
	1.0328	−430.6	1.0754	−339.6
April 2024	1.0702	−490.6	1.0783	−447.1
July 2024	1.0810	−713.8	1.0880	−498.0

## Data Availability

The raw data supporting the conclusions of this article will be made available by the authors on request.
